# Paclitaxel combined with Compound K inducing pyroptosis of non-small cell lung cancer cells by regulating Treg/Th17 balance

**DOI:** 10.1186/s13020-024-00904-2

**Published:** 2024-02-15

**Authors:** Hongzheng Wang, Min Huang, Mengyuan Zhu, Chi Su, Yijian Zhang, Hongyu Chen, Yuexin Jiang, Haidi Wang, Qinglong Guo, Shuai Zhang

**Affiliations:** 1 State Key Laboratory of Natural Medicines, Jiangsu Key Laboratory of Carcinogenesis and Intervention, China Pharmaceutical University, 24 Tongjiaxiang, Nanjing, 210009 People’s Republic of China; 2 Jiangsu Cancer Hospital & Jiangsu Institute of Cancer Research & The Affiliated Cancer Hospital of Nanjing Medical University, Nanjing, 21009 People’s Republic of China; 3 Jiangsu Key Laboratory of Molecular and Translational Cancer Research, Nanjing, 210009 People’s Republic of China; 4 The Fourth Clinical College of Nanjing Medical University, Nanjing, 210009 People’s Republic of China

## Abstract

**Background:**

Immune checkpoint inhibitors, which have attracted much attention in recent years, have achieved good efficacy, but their use is limited by the high incidence of acquired drug resistance. Therefore, there is an urgent need to develop new immunotherapy drugs. Compound taxus chinensis capsule (CTC) is an oral paclitaxel compound drug, clinical results showed it can change the number of regulatory T cells and T helper cell 17 in peripheral blood. Regulating the balance between regulatory T cells and T helper cell 17 is considered to be an effective anticancer strategy. Paclitaxel and ginsenoside metabolite compound K are the main immunomodulatory components, it is not clear that paclitaxel combined with compound K can inhibit tumor development by regulating the balance between regulatory T cell and T helper cell 17.

**Methods:**

MTT, EdU proliferation and plate colony formation assay were used to determine the concentration of paclitaxel and compound K. AnnexinV-FITC/PI staining, ELISA, Western Blot assay, Flow Cytometry and Immunofluorescence were used to investigate the effect of paclitaxel combined with compound K on Lewis cell cultured alone or co-cultured with splenic lymphocyte. Finally, transplanted tumor C57BL/6 mice model was constructed to investigate the anti-cancer effect in vivo.

**Results:**

According to the results of MTT, EdU proliferation and plate colony formation assay, paclitaxel (10 nM) and compound K (60 μM) was used to explore the mechanism. The results of Flow Cytometry demonstrated that paclitaxel combined with compound K increased the number of T helper cell 17 and decreased the number of regulatory T cells, which induced pyroptosis of cancer cells. The balance was mediated by the JAK–STAT pathway according to the results of Western Blot and Immunofluorescence. Finally, the in vivo results showed that paclitaxel combined with compound K significantly inhibit the progression of lung cancer.

**Conclusions:**

In this study, we found that paclitaxel combined with compound K can activate CD8^+^ T cells and induce pyroptosis of tumor cells by regulating the balance between regulatory T cells and T helper cell 17. These results demonstrated that this is a feasible treatment strategy for lung cancer.

## Introduction

According to the data released by World Health Organization, there were 9.96 million cancer deaths worldwide in 2020, of which China accounted for 30%. Lung cancer ranked first in mortality [1]. And 127,000 Americans are expected to die from lung cancer in 2023 [2]. Because the early symptoms of lung cancer are easy to be ignored, it often develops to the middle and late stages when diagnosed, so lung cancer has a high mortality rate [3]. The main treatments for non-small cell lung cancer (NSCLC) patients include surgery, radiotherapy, and drug therapy [4]. Although these methods used alone or in combination have certain effects, they still have great limitations [5]. Compared with the above-mentioned therapies, immunotherapy also has good efficacy and can significantly prolong the survival time [6]. Among them, immune checkpoint inhibitors (ICIs) targeting PD-1/PD-L1 are the most widely used, however, this therapy is only suitable for people with high PD-1/PD-L1 expression [7]. In addition, studies have shown the emergence of acquired resistance to ICIs treatment [8]. Therefore, there is an urgent need to develop new immunotherapy drugs as an alternative to ICIs therapy.

There are a large number of immunosuppressive cells in the tumor microenvironment (TME), such as myeloid-derived immunosuppressive cells (MDSCs), tumor-associated macrophages (TAMs) and regulatory T cells (Tregs), which significantly inhibit the infiltration and function of cytotoxic T lymphocytes (CTL), resulting in acquired drug resistance [9]. In 1970, Treg was discovered as a member of immunosuppressive cells in TME, forkhead box protein P3 (Foxp3) is the most specific marker of naturally CD4^+^CD25^+^ Tregs (nTregs) [10, 11]. The proportion of CD4^+^ Foxp3^+^Treg cells is significantly increased in patients with advanced NSCLC. The percentage of Treg cells is negatively correlated with the level of Interleukin-17 (IL-17), but positively correlated with IL-10 [12]. IL-17 is mainly expressed in T helper cell 17 (Th17), while IL-10 is mainly expressed in Treg, and there is a balance between Treg and Th17 [13]. The ratio of Foxp3 and Th17 marker protein-retinoic acid receptor-related orphan receptor γ (RORγt) in peripheral blood of NSCLC patients is higher than that of normal people, suggesting that the imbalance of Treg/Th17 is related to the development of NSCLC [14].

Naive CD4^+^ T cells are regulated by different cytokines and differentiate into Th1, Th2, Th17 or Treg. On the one hand, Th1 differentiation can be activated by Interferon-γ (IFN-γ) and IL-2, while Th2 differentiation be activated by IL-4. On the other hand, Th1 exert an anti-tumor immune response by secreting cytokines such as TNF-α and IL-2, and in contrast to Th1, Th2 can suppress the immune system and promote tumor progression [15].Besides, Naive CD4^+^ T cells differentiate into Th17 cells in the presence of IL-6 and TGF-β, and convert into Treg cells in the presence of IL-2 and TGF-β [16, 17]. Different cytokines can induce Naive CD4 ^+^ T cells to differentiate into different subtypes by activating Janus kinase (JAK) signal transducers and activators of transcription (STAT) pathway [18]. IL-6 activates JAK2–STAT3 signal transduction to induce RORγt transcription, while IL-2 activates JAK1/3–STAT5 pathway to induce Foxp3 transcription, thereby regulating differentiation direction [16, 17, 19].

Throughout the history of drug discovery, natural products and their derivatives have contributed greatly to cancer therapy [20]. Ginsenoside is one of the main active components of ginseng, which has a unique role in immune regulation [21]. Ginsenoside Compound K (CK) and Rh2 are the main metabolites of ginsenoside polysaccharide. Both compounds have high oral bioavailability. Many studies have explained the immunomodulatory function of Rh2, but few studies on CK [22, 23]. Paclitaxel (PTX) is a first-line chemotherapy drug for advanced NSCLC. Although it has good efficacy, drug resistance and high cytotoxicity limit its use [24]. Some studies have shown that PTX can selectively reduce Treg in the peripheral blood of NSCLC patients, while promoting the production of IFN-γ and IL-2 and the activation of CD8^+^T cells [25]. We hypothesized that CK, like Rh2, also has an immunomodulatory function, which can increase the efficacy of PTX and reduce the side effects of PTX by reducing the dose of PTX. In addition, we also investigated the regulatory effect of the combination of the two drugs on Treg/Th17 balance, which may provide a new therapeutic idea for NSCLC treatment.

## Materials and methods

### Compounds and reagents

CK (C_36_H_61_O_8_; MW = 621.88) was a white solid powder with a purity of 98% purchased from DILGER MEDICAL (Nanjing, China). For in vitro experiments, CK was dissolved with dimethyl sulfoxide (DMSO, 276855-100ML, Sigma-Aldrich, Missouri, USA) into a 0.1 M stock solution and stored at − 80 °C. For in vivo experiments, CK was prepared into a suspension with 0.5% carboxymethylcellulose sodium at the desired concentration for oral administration. PTX (C_47_H_51_NO_14_; MW = 853.92) was a faint yellow power with a purity of 98% purchased from Lvye Pharma (Nanjing, China). For in vitro experiments, PTX was dissolved with 5% glucose injection into a 0.1 mM stock solution and stored at − 80 °C. For in vivo experiments, PTX was dissolved with 5% glucose injection (DCPC, Beijing, China) to the desired concentration to facilitate intraperitoneal administration. As for other reagents, SH-4-54 (HY-16975) and Z-VAD-FMK (HY-16658B) were obtained from MCE (New Jersey, USA), D-Mannose was obtained from CSNpharm (CSN23272, Chicago, USA). Antibodies Caspase1 (A0964), GSDMD (A18281), GSDMD-N (A20197), Caspase3 (A2156), Cleaved-Caspase3 (A19654), GSDME (A7432), JAK1 (A18323), p-JAK1 (AP0530), JAK2 (A19629), p-JAK2 (AP0531), STAT3 (A1192), STAT5 (A5029), Granzyme B (A2557), Perforin 1 (A0093), LaminA/C (A0249) and β-actin (AC026) were purchased from Abclonal (Wuhan, China). IL-6R (ab271042), p-STAT3 (ab267373) and p-STAT5 were purchased from Abcam (Cambridge, UK). RORγt (609051), CD3 (100201) and CD28 (102101) were purchased from Biolegend (California, USA). CD3-PE (E-AB-F1013D), CD3-FITC (E-AB-F1013UC), CD4-FITC (E-AB-F1097C), CD8-APC (E-AB-F1104E), IL-17A-APC (E-AB-F1199E), CD25-PE (E-AB-F1102D), CD196-APC (E-AB-F1158E) and Foxp3-APC (E-AB-F1238E) were purchased from Elabscience (Wuhan, China). RORγt-PE (563081), IL-10-BV65 (564083), CD45-APC-Cy^TM^7 (557659) and CD4-PerCP-Cy^TM^5.5 (550954) were purchased from Becton, Dickinson and Company (New Jersey, USA).

### Cell culture

The cell lines A549, Lewis and 293T were purchased from the cell bank of Shanghai Institute of Biochemistry and Cell Biology. Peripheral blood mononuclear cells (PBMCs) from healthy donors (The Affiliated Cancer Hospital of Nanjing Medical University, Nanjing, China) were collected using lymphocyte-monocyte separation medium (Jingmei, Nanjing, China). Mouse spleen lymphocytes (SPC) were obtained from normal male C57BL/6 mice aged 6–8 weeks (Slaccas Shanghai Laboratory Animal Co, Shanghai, China). All cells were passaged and expanded less than 15 times, stored at liquid nitrogen. Lewis, A549, 293T and SPC were cultured in DMEM medium (Gibco, California, USA), with 10% heat-inactivated fetal bovine serum (Wisent Bio Products, Canada), 100 U/mL benzylpenicillin, and 100 U/mL streptomycin. PBMC were cultured in X-VIVO serum-free medium (Lonza, Maryland, USA). All cells were cultured in a carbon dioxide incubator (3311, ThermoFisher, Massachusetts, USA) with 5% CO_2_, 37 °C, and suitable humidity.

All experiments using animal samples were approved by Institutional Animal Care and Use Committee of NMU, and all healthy donors were provided informed consent. Ethics number of animal experiments: IACUC-2103045.

### The extraction and isolation of SPC

Mouse spleens were cut into small pieces and placed on a cell sieve, and cells were collected with phosphate-buffered saline (PBS) after grinding several times. The cells were centrifuged at 3000 rpm for 5 min before red blood cell lysate was added, placed at 4 °C for 5 min, and spleen cells were obtained after washing with PBS. In the co-culture system, SPC were added to the co-culture chambers (PICM03050, Merck, New Jersey, USA).

### Cell viability assay

Lewis, A549, 293T and PBMCs were seeded with appropriate density in 96-well plates. The cells were treated with different concentrations of PTX or CK for 48 h. 20 μL MTT (CSN12440, CSNpharm) were added to each well and incubated at 37 °C for 4 h. and 100 μL DMSO was added to each well to dissolve the Formazan crystals formed at the bottom of the 96-well plate. The optical absorbance was measured at 570 nm with a SynergyTM HT multi-mode reader (Bio-Tek, Winoosky, VT, USA). The cell viability inhibition rate was calculated using the following formula:

$${\text{Inhibition rate}}\left( \% \right) = \left[ {{\text{OD}}\left( {\text{control group}} \right) - {\text{OD}}\left( {\text{treatment group}} \right)} \right]/{\text{OD }}\left( {\text{control group}} \right) \, \times {1}00\%$$

The IC50 value refers to the concentration of LW-213 which can inhibit cell viability by 50% and is calculated using the logit method.

### Edu image assay

Cells were treated with different concentrations of PTX or CK for 48 h. After treatment, the cells were stained with Edu and processed according to the manufacturer’s instruction (KGA331-100, KeyGEN BioTECH, Nanjing, China). Finally, the cells were blocked with DAPI (P0131, Beyotime Biotechnology, Shanghai, China) and observed by fluorescence microscope (Axio Scope.A1, Zeiss, Oberkochen, Germany).

### Plate clone formation assay

Cells were treated with different concentrations of PTX or CK for 48 h. After treatment, the cells were stained with 0.1% crystal violet staining solution (46364-250MG, Sigma-Aldrich). After staining, photographs were taken and clones were counted.

### Cell apoptosis

LLC cells were collected and stained with Annexin V and PI according to the instruction of Annexin V-PI Apoptosis Detection Kit (A211-01/02, Vazyme Biotec, Nanjing, China). The fluorescence intensity was detected by BD Accuri™ C6 Plus Flow Cytometer (660517, Becton, Dickinson and Company) and the data analysis was performed by Flowjo software (Tree Star, Ashland, OR, USA).

### Membrane surface antigen detection

Cells were treated with PTX or CK or both for 48 h. After treatment, the cells were collected and blocked with 0.5% Bovine Serum Albumin (BSA, A3858-100G, Sigma-Aldrich) for 20 min before labelled with fluorescent-conjugated antibody. Then cells were washed by PBS with 0.5% BSA for two times. Finally, the cells were resuspended in PBS and detected by flow cytometer.

### Intracellular antigen detection

Cells were collected and treated with Transcription Factor Fix/Perm buffer (562574, Becton, Dickinson and Company) for 50 min away from light. Then the cells were washed by Transcription Factor Wash/Perm buffer and labelled with fluorescent-conjugated antibody. After washed by Wash buffer for two times, the cells were resuspended in PBS and detected by flow cytometer. When the intracellular levels of IL-10 and IL-17A were assayed, the cells were simultaneously incubated with 1X protein transport inhibitor cocktail (00-4980-03, ThermoFisher) for 48 h.

### Detection of tumor infiltrating lymphocytes

The tumor tissues were cut into small pieces and then digested with 1 mg/ml collagenase IV (A004186, Sangon Biotech, Nanjign, China), 20 μg/ml Hyaluronidase (A002594, Sangon Biotech) and 60 μg/ml Deoxyribonuclease I (A610099, Sangon Biotech) for 1 h at 37 °C. Afterwards, an equal amount of medium was added to terminate the digestion, and single cell suspensions were obtained by repeatedly blowing and placing into a cell sieve, tumor infiltrating lymphocytes were then detected by BD FACSCelesta™ Flow Cytometer (660344, Becton, Dickinson and Company). Due to the complexity of the tumor tissue, both Th17 cells and Treg cells were analyzed in the CD45^+^CD4^+^ cell population, while CD4^+^CD8^−^ cells and CD4^−^CD8^+^ cells were analyzed in the CD45^+^CD3^+^ cell population.

### ELISA assay

Cells were treated with PTX or CK or both for 48 h. After treatment, the expression of related-cytokines was determined by Elisa Kit according to the instructions.

### Western blotting (WB)

Whole proteins were extracted by RIPA buffer (89901, ThermoFisher,). Nuclear and Cytoplasminc proteins were extracted according to the instructions of Nuclear and Cytoplasmic Protein Extraction Kit (KGP150, KeyGEN, Nanjing, China). The concentration was determined by BCA Protein Assay Kit (23227, ThermoFisher). The proteins were separated on SDS-PAGE gels and transferred to NC membrane. After incubated with 3% BSA for 1 h, the target bands were incubated with the corresponding primary antibody and homologous secondary antibody. Finally, the bands were enhanced by ECL system (36222ES60, YEASEN, Shanghai, China) and visualized by Amersham Imager 600 (General Electric Company, Schenectady, New York, USA).

### Immunofluorescence (IF)

The SPC in co-culture system were treated with PTX or CK or both for 48 h. After treatment, the cells were collected and smeared on the cover glass. Then the cells were fixed with ice-cold 4% paraformaldehyde for 15 min and permeabilized with 0.2% Triton X-100 (X100-500 ML, Sigma-Aldrich) for 30 min. After blocked with 3% BSA at 37℃ for 1 h, the cells were labelled with primary antibody overnight at 4 °C, followed by incubated with Alexa Fluor secondary antibody (A-11010, ThermoFisher) for 1 h. Finally, the cells were incubated with DAPI and observed by laser confocal microscope (Fluoview FV1000, Olympus, Tokyo, Japan).

### Immunohistochemistry

The tumor samples were fixed with formalin and followed by paraffin embedding and sectioning. After deparaffinization and antigen repair, the samples were processed for subsequent operation according the protocol of IHC Prep &Detect Kit for Rabbit Primary Antibody (PK10017, Proteintec, Wuhan, China). Finally, the samples were observed by fluorescence microscope.

### Xenografts assay

Female C57BL/6 mice aged 6–8 weeks were used in the experiment, and 6 × 10^5^ Lewis cells were inoculated in the right leg of mice. When tumor volume reached 100 mm^3^, the mice were divided into five groups (Blank, Ctrl, PTX, CK, PTX + CK), every group has five mice. PTX (10 mg/kg), CK (40 mg/mg), PTX (10 mg/kg) + CK (40 mg/mg) were administrated daily. Tumor volumes were recorded every two days and the tumor volume (mm^3^) was calculated as Eq:

$${\text{V}}_{{{\text{Tumor}}}} = \, \left( {\text{Shortest diameter}} \right)^{{2}} \times \left( {\text{Longest diameter}} \right)/{2}.$$

Tumor tissues were sectioned and processed for Immunohistochemistry and protein extraction to detect the distribution and expression of targeted proteins. Spleen tissues were used to detect the balance of Treg/Th17.

### Statistical analysis

All experimental data were expressed as mean ± Standard Deviation (SD) and at least three independent experiments performed in a parallel manner. Statistical comparisons between multiple groups were compared using one-way ANOVAs with the Dunnett post-hoc test. A *P* < 0.05 was considered statistically significant (*P < 0.05, **P < 0.01, ***P < 0.001, ****P < 0.0001).

## Results

### The combination of PTX and CK can significantly inhibit the viability of NSCLC cells

**Fig. 1:**
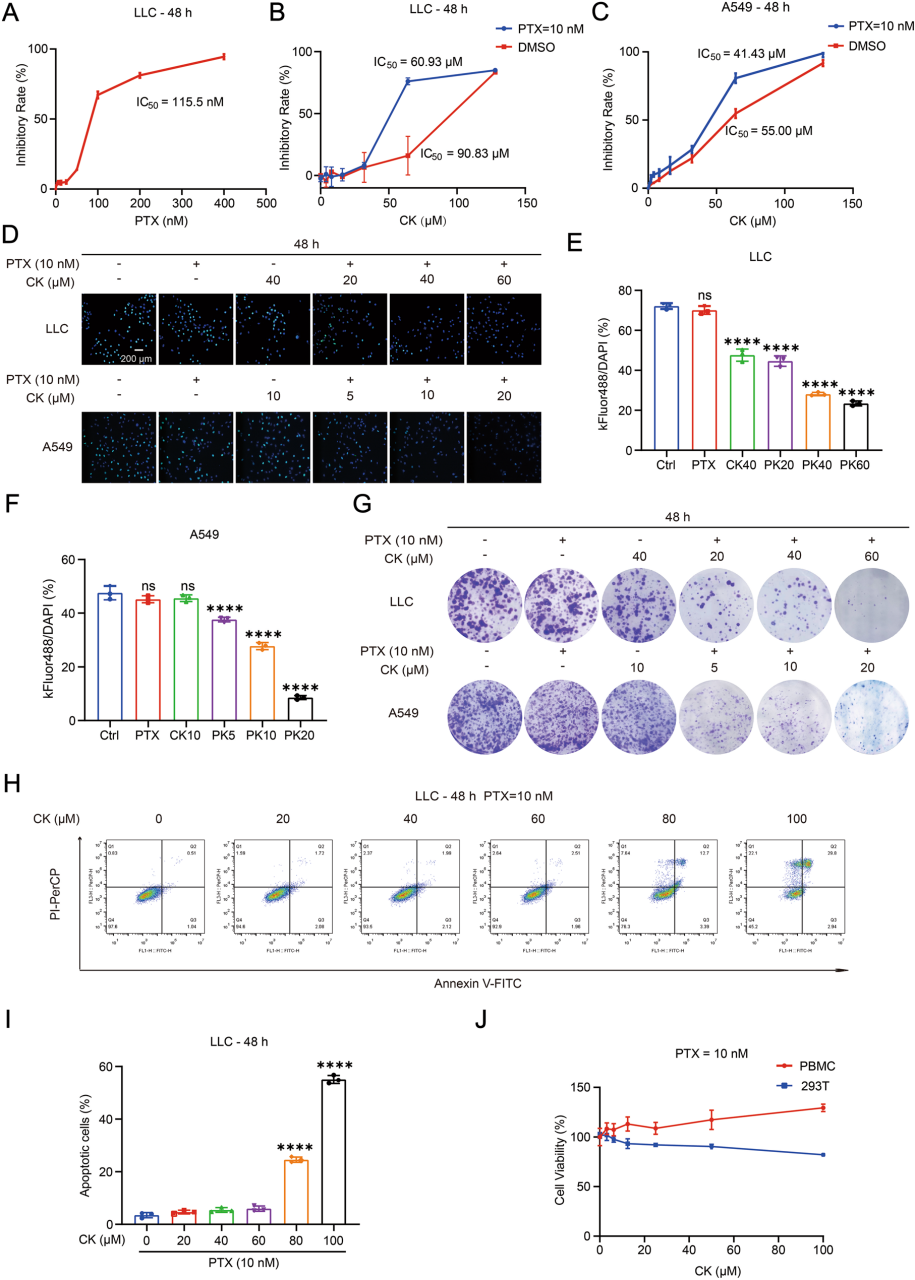
The combination of PTX and CK can significantly inhibit the viability of NSCLC cells. **A** The Lewis cells were treated with PTX for 48 h, the growth inhibition effect was assessed by MTT assay. **B** The Lewis cells were treated with CK or CK combined with 10 nM PTX for 48 h, the growth inhibition effect was assessed by MTT assay. **C** The A549 cells were treated with CK alone or CK combined with 10 nM PTX for 48 h, the growth inhibition effect was assessed by MTT assay. **D**–**F** The Lewis and A549 cells treated with PTX, CK or CK combined with 10 nM PTX for 48 h were incubated with EdU for the detection of cell viability by fluorescence microscope. **G** The Lewis and A549 cells treated with PTX, CK or CK combined with 10 nM PTX for 48 h were stained by crystal violet solution, the cell viability was represented by colony forming efficiency. **H**, **I** The Lewis cells were treated with CK combined with 10 nM PTX for 48 h, apoptotic cells (%) were detected by Flow Cytometry. **J** The PBMC and 293T cells were treated with CK combined with 10 nM PTX for 48 h, the cell viability was assessed by MTT assay. Data are mean ± SD for ≥ 3 independent experiments; ***P < 0.001, ****P < 0.0001, vs Ctrl or CK (0 μM)

The use of non-killing concentrations of PTX and CK was required in subsequent experiments, so we first screened the concentrations. As Fig. 1A shown, the IC_50_ values of PTX on Lewis lung cancer cells (LLC) at 48 h is 115.5 nM, 10 nM PTX was used in in vitro experiments. Similarly, the IC_50_ of CK on LLC or A549 cells were obtained when treated alone or in combination with PTX (Fig. 1B, C). We observed that PTX combined with CK (PK) had significant inhibitory effect on the proliferation of LLC or A549 cells in a CK concentration-dependent manner (Fig. 1D–G). We further confirmed CK (60 μM)-maximum nonkilling concentration for subsequent cocultured experiments by Annexin-PI staining (Fig. 1H, I). Interestingly, PK had no significant effect on normal cells, demonstrating the safety of PK (Fig. 1J).

### The combination of PTX and CK can regulate the balance of Treg and Th17

**Fig. 2:**
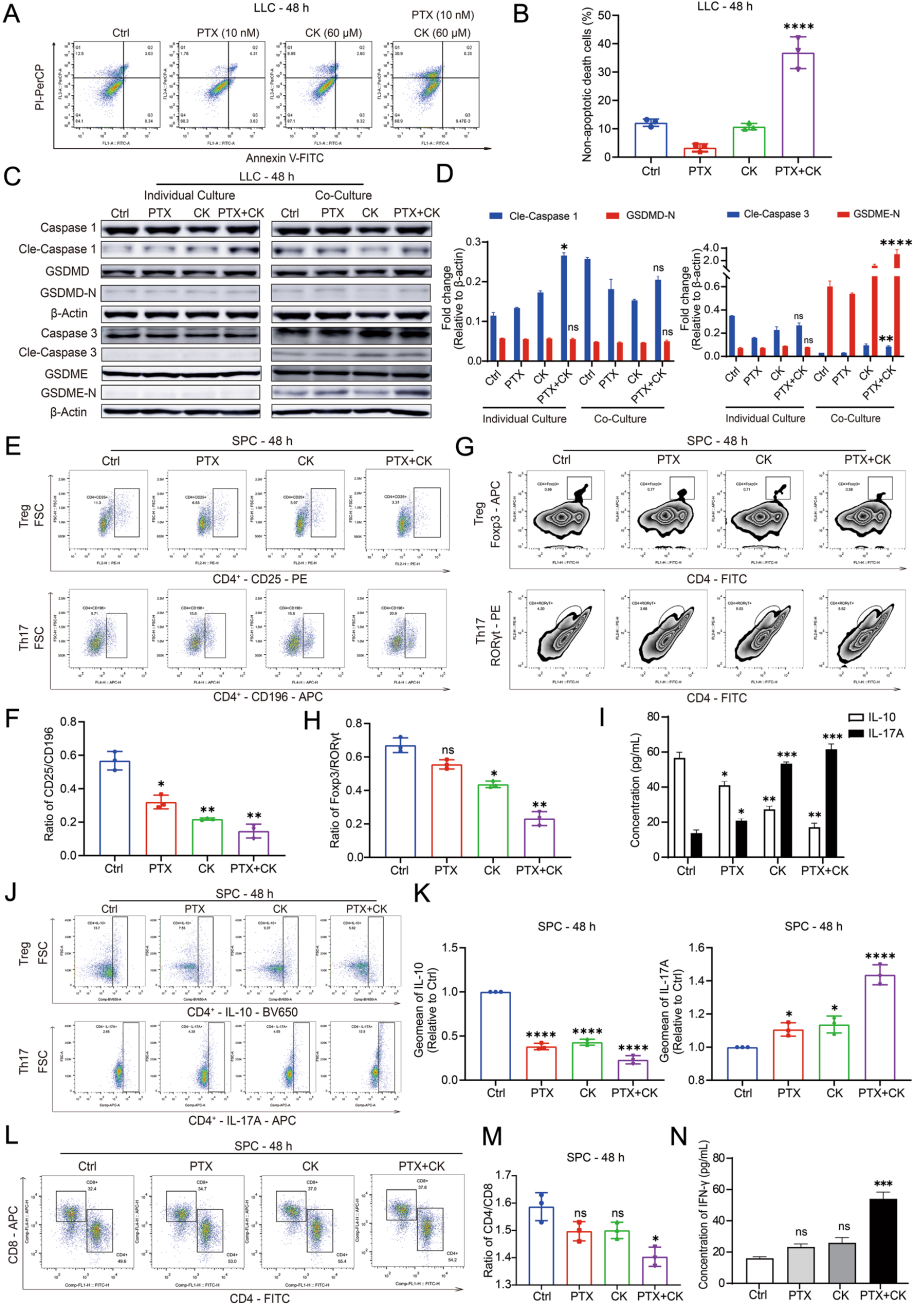
The combination of PTX and CK can regulate the balance of Treg and Th17. **A**, **B** The Lewis cells cocultured with SPC were treated with 60 μM CK combined with 10 nM PTX for 48 h, apoptotic cells (%) and non-apoptotic death cells were detected by Flow Cytometry. **C**, **D** The Lewis cells cultured alone or cocultured with SPC were treated with 60 μM CK, 10 nM PTX, or both for 48 h, the expression of pyroptosis-related proteins was detected by Western blot, β-actin was used as loading control. **E**, **F** The Lewis cells were cocultured with SPC and treated with 60 μM CK, 10 nM PTX, or both for 48 h. CD4^+^CD25^+^ cells and CD4^+^CD196^+^ cells in CD4^+^SPC were determined by Flow Cytometry, and the ratio of CD4^+^CD25^+^ cells to CD4^+^CD25^+^ cells was calculated. **G**, **H** The Lewis cells were cocultured with SPC and treated with 60 μM CK, 10 nM PTX, or both for 48 h. Foxp3^+^ cells and RORγt^+^ cells in SPC were detected by Flow Cytometry, and the ratio of Foxp3^+^ cells to RORγt^+^ cells was calculated. **I** The Lewis cells were cocultured with SPC and treated with 60 μM CK, 10 nM PTX, or both for 48 h. The concentration of IL-10 and IL-17A in co-culture system was determined by ELISA. **J** The Lewis cells were cocultured with SPC and 1X protein transport inhibitor cocktail and treated with 60 μM CK, 10 nM PTX, or both for 48 h. the IL-17A^+^ cells or IL-10^+^ cells in CD3^+^CD4^+^ cell population were determined by Flow Cytometry. **K** The relative Geomean of IL-17A or IL-10 to ctrl group. **L**, **M** The Lewis cells were cocultured with SPC and treated with 60 μM CK, 10 nM PTX, or both for 48 h. CD3^+^CD4^+^ cells and CD3^+^CD8^+^ cells in SPC were determined by Flow Cytometry, and the ratio of CD3^+^CD4^+^ cells to CD3^+^CD8^+^ cells was calculated. **N** The Lewis cells were cocultured with SPC and treated with 60 μM CK, 10 nM PTX, or both for 48 h. The concentration of IFN-γ in co-culture system was determined by ELISA. Data are mean ± SD for ≥ 3 independent experiments; *P < 0.05, **P < 0.01, ***P < 0.001, ****P < 0.0001, vs Ctrl

Previous studies have shown that PTX can reduce the number of Treg and CK can regulate the balance of Treg/Th17 [26, 27]. Therefore, we next explored whether PK could affect Treg/Th17 balance and regulate immune function. To mimic the tumor immune microenvironment in vitro, SPC was cocultured with LLC and co-treated with drugs. We first examined whether non-killing concentrations of PK could induce LLC cell death in co-culture system, and the results showed that PK could induce the non-apoptotic death of LLC cells (Fig. 2A, B). Based on the distribution of dead cells, we hypothesized that PK induced pyroptosis in LLC. Classical pyroptosis depends on the function of the Caspase1-GSDMD pathway [28, 29]. In recent years, it has been found that the Caspase3-GSDME pathway can also induce pyroptosis [30]. According to WB, we speculated that PK can induce pyroptosis by activating the Caspase3-GSDME pathway (Fig. 2C, D). Since no pyroptosis occurred in LLC cultured alone, suggesting that immune cells contributed to pyroptosis, we examined the regulation of Treg/Th17 balance. As speculated, we observed an increase in Th17 and decrease in Treg by specific antigen assays (Fig. 2E–H). Furthermore, the increased secretion of IL-17A and the decreased secretion of IL-10 supported this conclusion (Fig. 2I), and we also blocked the secretion of both and found that IL-17 was upregulated and IL-10 was downregulated in SPC treated by PK (Fig. 2J, K). Previous studies have shown that RORγt regulated the immune microenvironment by promoting tumor infiltration of CTL [31], and CTL induced tumor cell death by secreting IFN-γ [32, 33]. Predictably, the proportion of CTL and release of IFN-γ also increased by PK (Fig. 2L–N). These findings demonstrated that PK induced LLC pyroptosis by regulating Treg/Th17 balance.

### The combination of PTX and CK can affect JAK–STAT pathway

**Fig. 3:**
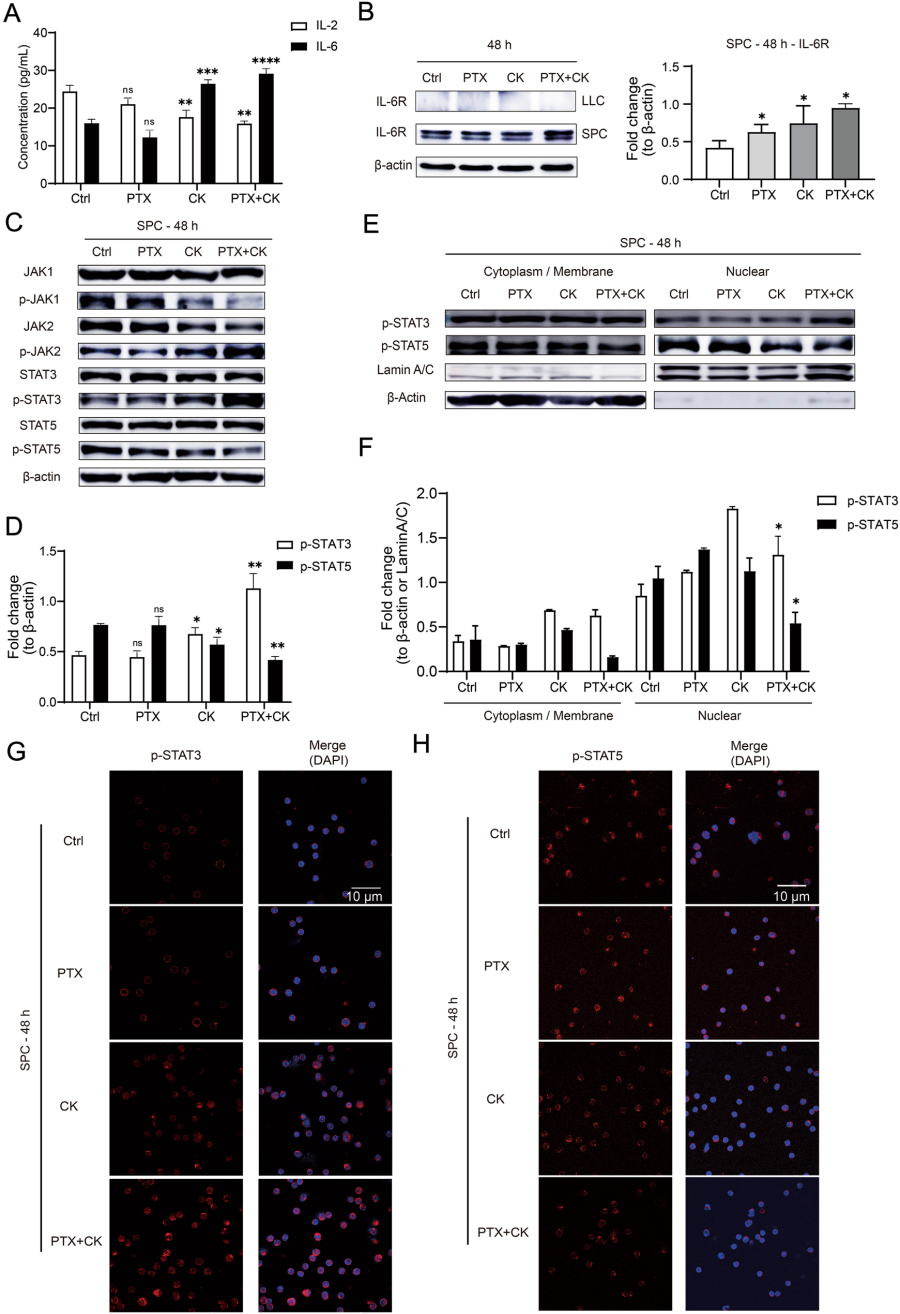
The combination of PTX and CK can affect JAK–STAT pathway. The Lewis cells were cocultured with SPC and treated with 60 μM CK, 10 nM PTX, or both for 48 h. All experiments were carried out in the co-culture system, including Lewis cells, SPC or the whole system. **A** The concentration of IL-2 and IL-6 in co-culture system was determined by ELISA. **B** The expression of IL-6R in Lewis cells and SPC was detected by Westren blot, β-actin was used as loading control. **C**, **D** The expression of JAK1/2, p-JAK1/2, STAT3/5 and p-STAT3/5 in SPC was detected by Western blot, β-actin was used as loading control. **E**, **F** The levels of p-STAT3 and p-STAT5 in both cytoplasm/membrane and nuclei fraction of SPC were determined by Western blot, β-actin and LaminA/C were used as loading control. **G** The immunofluorescence analysis performed with anti-p-STAT3 (red) and DAPI, Scale bar: 10 μm. **H** The immunofluorescence analysis performed with anti-p-STAT5 (red) and DAPI, Scale bar: 10 μm. Data are mean ± SD for ≥ 3 independent experiments; *P < 0.05, **P < 0.01, ***P < 0.001, ****P < 0.0001, vs Ctrl

Treg and Th17 are derived from peripheral Naive CD4+ T cells [34], the differentiation of Naïve CD4^+^T cell is regulated by different cytokines, including IL-2, IL-6, TGF-β and so on [16, 17, 35]. PK administration for 48 h could significantly increase the expression of IL-6 and IL-6R in co-culture system, while decreasing the content of IL-2 (Fig. 3A, B). It has been shown that IL-2 and IL-6 regulate the differentiation process mainly by activating JAK–STAT signal transduction, including IL-2-JAK1/3-STAT5 and IL-6-JAK2-STAT3 pathways [16–18]. Since PK affected IL-2 and IL-6 concentrations in the co-culture system, we next examined whether PK affected the JAK–STAT pathway in SPC. As expected, PK upregulated the expression of phospho-JAK2 (p-JAK2) and phospho-STAT3 (p-STAT3), meanwhile, deregulated the expression of phospho-JAK1 (p-JAK1) and phospho-STAT5 (p-STAT5) (Fig. 3C, D). Furthermore, we found that PK induced the translocation of p-STAT3 and inhibited the translocation of p-STAT5 from cytoplasm to nucleus (Fig. 3E–H). These results described above showed PK could regulate the activation of JAK–STAT pathway by affecting the expression of IL-2 and IL-6.

### The JAK–STAT pathway plays a key role in the regulation of Treg/Th17 balance

**Fig. 4:**
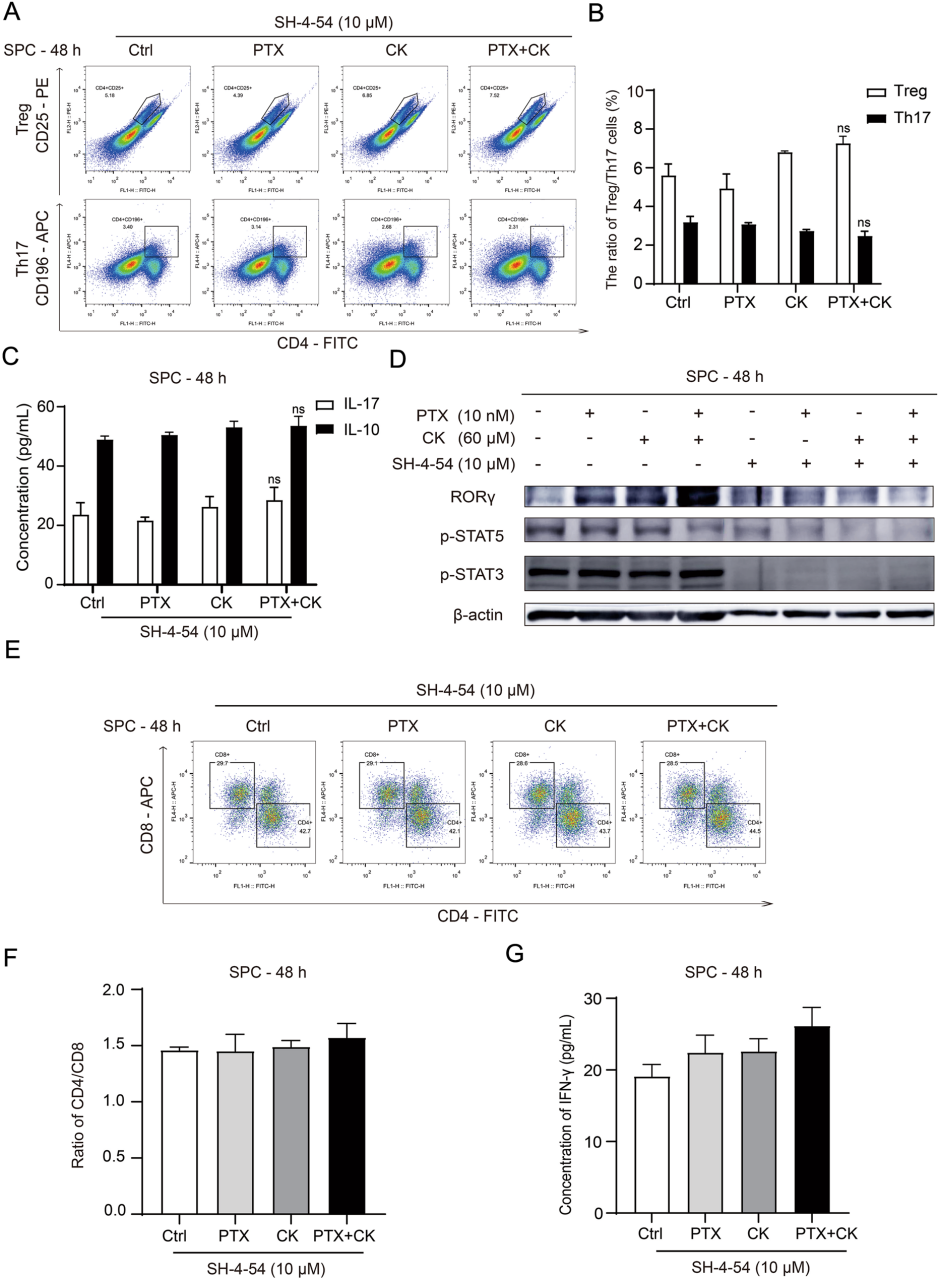
The JAK–STAT pathway plays a key role in the regulation of Treg/Th17 balance. The Lewis cells were cocultured with SPC and treated with 60 μM CK, 10 nM PTX, or both for 48 h. All experiments were carried out in the co-culture system, including Lewis cells, SPC or the whole system. **A**, **B** In addition to CK or PTX, the co-cultured system was cotreated with 10 μM SH-4-54 for 48 h. CD4^+^CD25^+^ cells and CD4^+^CD196^+^ cells in SPC were determined by Flow Cytometry, and the ratio of CD4^+^CD25^+^ cells to CD4^+^CD25^+^ cells was calculated. **C** In addition to CK or PTX, the co-culture system was cotreated with 10 μM SH-4-54 for 48 h. The concentration of IL-10 and IL-17A in co-culture system was determined by ELISA. **D** In addition to CK or PTX, the co-culture system was cotreated with or without 10 μM SH-4-54 for 48 h. The expression of RORγ, p-STAT3 and p-STAT5 was detected by Western blot. **E**, **F** In addition to CK or PTX, the co-culture system was cotreated with 10 μM SH-4-54 for 48 h. CD3^+^CD4^+^ cells and CD3^+^CD8^+^ cells in SPC were determined by Flow Cytometry, and the ratio of CD3^+^CD4^+^ cells to CD3^+^CD8^+^ cells was calculated. **G** In addition to CK or PTX, the co-culture system was cotreated with 10 μM SH-4-54 for 48 h. The concentration of IFN-γ in co-culture system was determined by ELISA. Data are mean ± SD for ≥ 3 independent experiments; *P* values have no significant difference, vs Ctrl

We next explored whether JAK–STAT pathway activation was necessary for the regulation of Treg/Th17 balance. SH-4-54, a specific inhibitor, prevents activation of STAT3 and STAT5 by inhibiting their phosphorylation [36, 37]. Therefore, we next tested whether JAK–STAT pathway was necessary by co-treatment with SH-4-54. We found that PK failed to change the proportion of Treg and Th17 after treatment with SH-4-54, nor did the concentrations of IL-10 and IL-17A in the co-culture system (Fig. 4A–C). As expected, phosphorylation of STAT3 and STAT5 was also inhibited by SH-4-54 (Fig. 4D). Moreover, the proportion of CTL and the concentration of IFN-γ did not change significantly under the treatment of SH-4-54 (Fig. 4E–G). These results demonstrated that the effects of PK relied on the activation of JAK2–STAT3 pathway.

### The combination of PTX and CK can induce pyroptosis of Lewis cells in the co-culture system

**Fig. 5:**
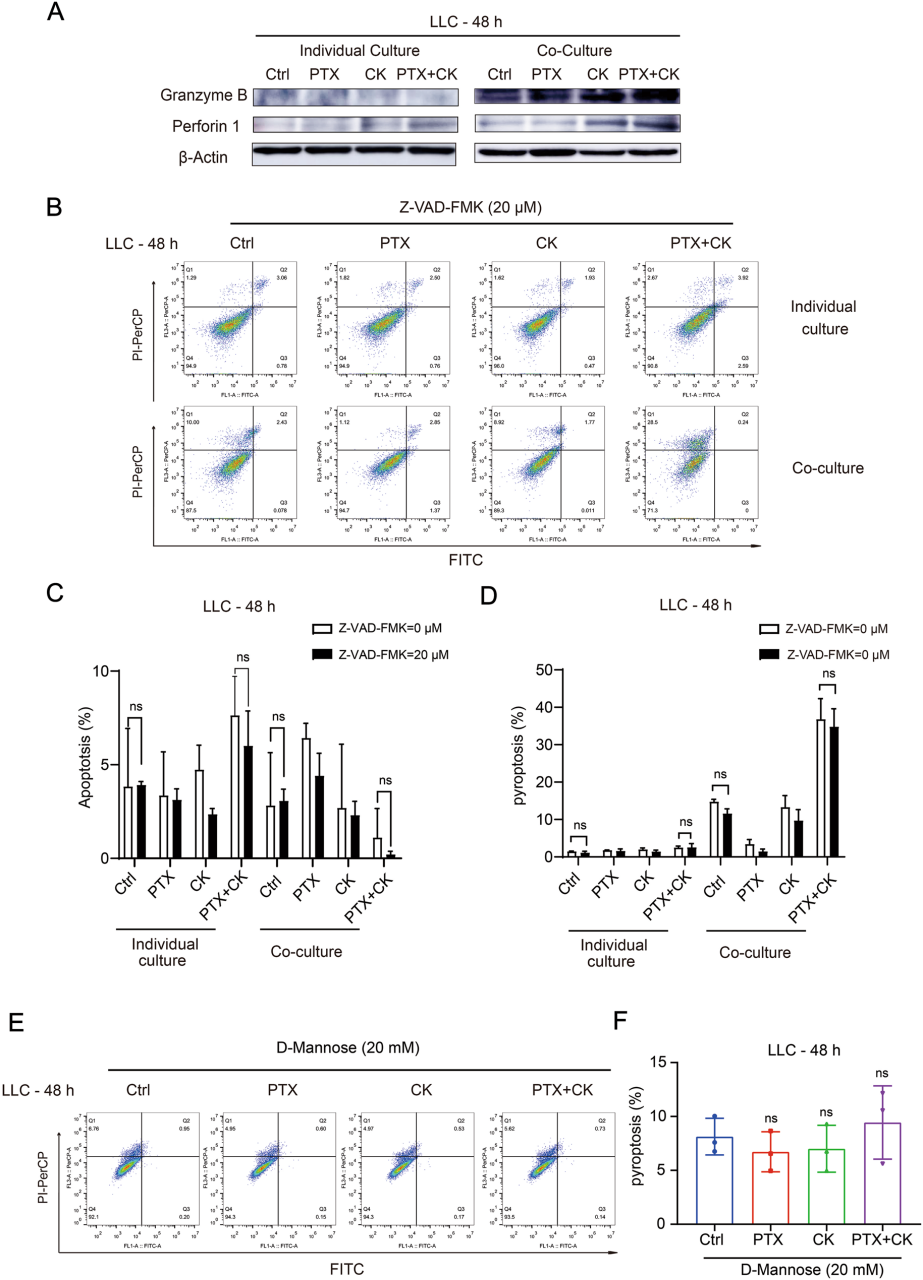
The combination of PTX and CK can induce pyroptosis of Lewis cells in the co-culture system. **A** Lewis cells cultured alone or in the co-culture system were treated with CK or PTX or both. The expression of Granzyme B and Perforin 1 in Lewis cells was detected by Western blot, β-actin was used as loading control. **B**–**D** Lewis cells cultured alone or in the co-culture system were pretreated with 20 μM Z-VAD-FMK for 1 h, and then treated with 60 μM CK, 10 nM PTX, or both for 48 h. Apoptotic cells (%) and non-apoptotic death cells (%) were detected by Flow Cytometry. **E**, **F** Lewis cells cultured alone or in the co-culture system were pretreated with 20 mM d-mannose for 3 h, and then treated with 60 μM CK, 10 nM PTX, or both for 48 h. Pyroptosis (%) was detected by Flow Cytometry. Data are mean ± SD for ≥ 3 independent experiments; *P* values have no significant difference, vs Ctrl

We next explored the molecular mechanism of PK-induced pyroptosis. CTL is one of the main effector cells to eliminate tumor cells, and its cytotoxicity depends on the granule exocytosis pathway [38]. In the granule exocytosis pathway, perforin 1 delivered Granzyme B (GZMB) into tumor cells and induced tumor cell death [39]. GZMB can activate GSDME through caspase3-dependent and caspase3-independent pathways, thereby initiating the pyroptosis process [30]. As Fig. 5A shown, PK significantly increased the expression of GZMB and perforin 1 in LLC cocultured with SPC. Pk-induced pyroptosis was further confirmed to be independent of Caspase3 by co-treated with Z-VAD-FMK (20 μM), a pan-caspase inhibitor (Fig. 5B–D), in addition, the results in the non-co-culture system showed that 20 μM Z-VAD-FMK in combination with PTX and CK could not cause death of LLC and was a safe concentration (Fig. 5B). In contrast, inhibition of GSDME cleavage by d-mannose markedly suppressed PK-induced pyroptosis [40] (Fig. 5E, F). These results described above showed that PK induced pyroptosis of LLC in the co-culture system in a caspase-3-independent but GSDME-dependent manner.

### The combination of PTX and CK can inhibit the growth of transplanted tumor in mice by activating immune function

**Fig. 6:**
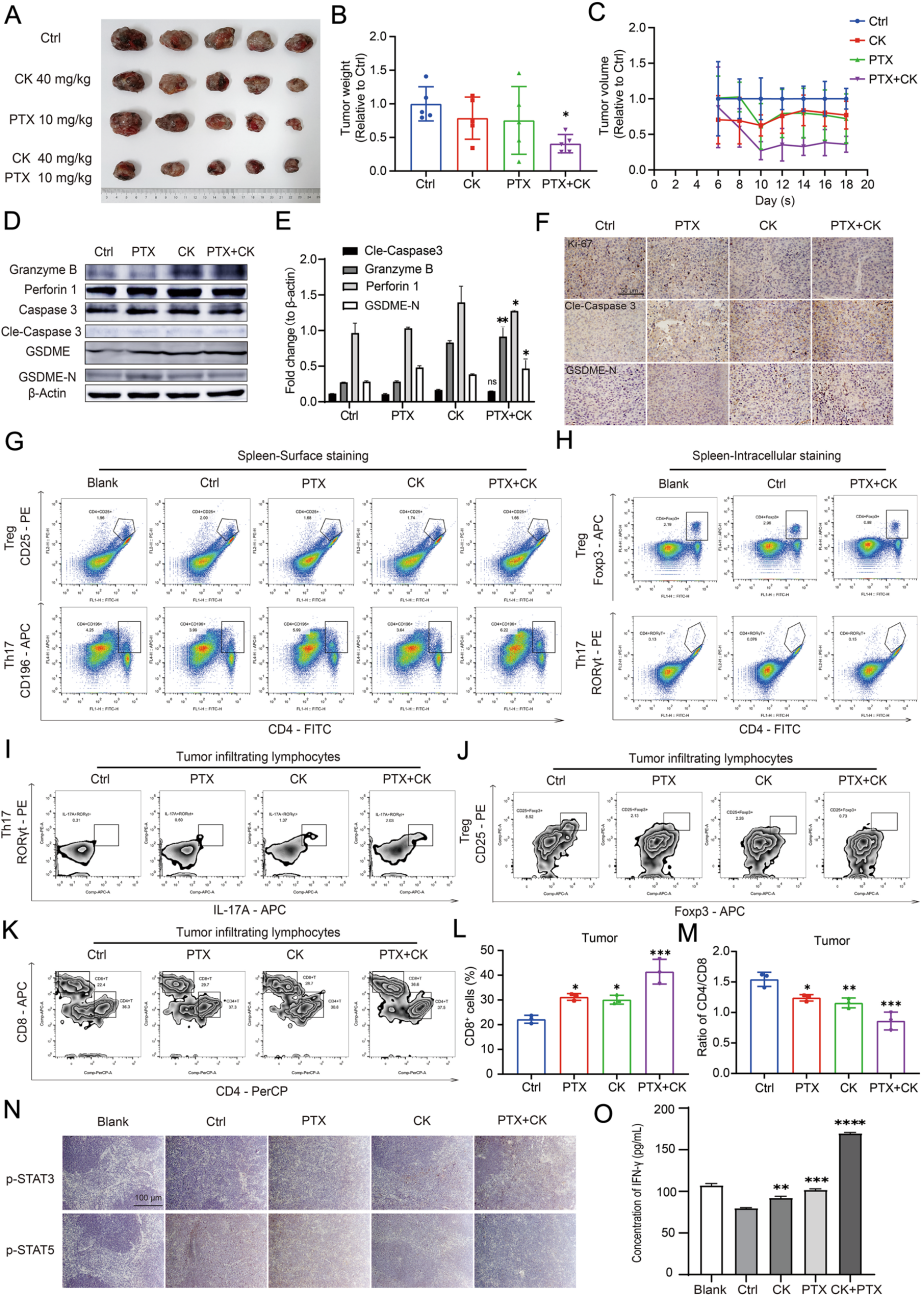
The combination of PTX and CK can inhibit the growth of transplanted tumor in mice by activating immune function. **A**–**C** Average tumor volume (A, B) and Average tumor weight (H) in Ctrl, CK, PTX and PTX + CK group. **D**, **E** The expression of Granzyme B, Perforin 1, Caspase-3, Cle-Caspase3, GSDME and GSDME-N in tumor cells from every group was determined by Western blot, β-actin was used as loading control. **F** The immunohistochemical analysis performed with anti-Ki67, anti-Cle-Caspase3 and anti-GSDME-N in tumor tissue, Scale bar: 50 μm. **G** The spleen cells were extracted from every group, CD4^+^CD25^+^ cells and CD4^+^CD196^+^ cells in SPC were determined by Flow Cytometry. **H** The spleen cells were extracted from Blank group, Ctrl group and PTX + CK group, Foxp3^+^ cells and RORγt^+^ cells in SPC were detected by Flow Cytometry. **I**, **J** The tumor infiltrating lymphocytes were extracted from every group, the RORγt^+^IL17A^+^ cells or CD25^+^Foxp3^+^ cells in CD45^+^CD4^+^ cell population were determined by Flow Cytometry. **K**–**M** The tumor infiltrating lymphocytes were extracted from every group, the CD4^+^ cells or CD8^+^ cells were determined by Flow Cytometry. **N** The immunohistochemical analysis performed with anti-p-STAT3 and anti-p-STAT5 in spleen tissue, Scale bar: 100 μm. **O** The concentration of IFN-γ in the blood samples from each group was determined by ELISA. Data are mean ± SD for ≥ 3 independent experiments; *P < 0.05, **P < 0.01, ***P < 0.001, ****P < 0.0001, vs Ctrl

We mimicked the tumor microenvironment by co-culturing SPC with LLC, and found that PK could induce pyroptosis in LLC by regulating Treg/Th17 balance. Based on these results, C57BL/6 mice LLC xenograft model was constructed to test whether PK could induce tumor cells pyroptosis in the mice model. The mice were inoculated subcutaneously with LLC and divided into different groups. Compared with Ctrl group, PK significantly inhibited tumor growth (Fig. 6A–C). Furthermore, the expression of pyroptosis-related proteins was increased by PK treatment (Fig. 6D, E). The upregulation of Cle-Caspase 3 was not obvious, possibly because tumor tissue proteins were not easy to be detected, so we verified it by immunohistochemistry (Fig. 6F). Studies have shown that the infiltration of Treg is increased in tumor tissues and blood system, and proportion of Treg is closely related to the prognosis of NSCLC patients [41]. Therefore, we examined the proportions of Treg and Th17 in spleens in mice model by specific antigen assay. Compared with Blank group, Treg increased and Th17 cells decreased significantly in Ctrl group (Fig. 6G, H). We observed that PTX,CK and PK increased the proportion of Th17 and decreased Treg, and the effect of PK was the most significant (Fig. 6G, H), this was consistent with the detection of tumor-infiltrating lymphocytes (Fig. 6I, J). It is noteworthy that the infiltration of CD8^+^ T cells was also significantly increased (Fig. 6K–M). In addition, the expression of p-STAT3 and p-STAT5 can be regulated by PK as in vitro (Fig. 6N). Similarly, the proportion of CTL in spleen and concentration of IFN-γ in peripheral blood was increased by PK treatment (Fig. 6O). Through a series of in vivo and in vitro experiments, we confirmed that PK could regulate Treg/Th17 balance, thereby regulating anti-tumor immunity and activating CTL-mediated immune response.

## Discussion

Lung cancer is the cancer with the highest mortality rate in the world. At the time of diagnosis, lung cancer patients tend to have advanced disease, and most of them are NSCLC. Radiotherapy and drug therapy are the main treatments for patients with advanced NSCLC in clinical practice [42], among which immunotherapy drugs have achieved good results, but there are also many shortcomings. In recent years, many biological immune agents have been developed and applied in clinical practice, but severe drug resistance limits their use [43–45]. In addition, immunosuppressive cells (such as Treg) in the tumor microenvironment can significantly inhibit CTL-mediated anti-tumor immunity [46–48], which is not conducive to the effect of immunotherapy drugs. There is a balance between Treg and Th17, and this balance is involved in the progression of many diseases [49–51]. In the peripheral blood of patients with advanced NSCLC, Treg/Th17 ratio is increased [52], and the number of Treg in lung adenocarcinoma was significantly higher than that in lung squamous cell carcinoma [53–55]. Therefore, we speculated that reducing this ratio can inhibit the disease progression of NSCLC.

Immune escape is an important mechanism in the occurrence and development of lung cancer. Lung cancer cells evade the recognition and attack of the immune system by changing the microenvironment, which is manifested as the imbalance between immune cells and cytokines [56]. Treg and Th17 cells are two T cell subsets differentiated by CD4^+^ naive T cells under the action of different cytokines. The regulation of Treg/Th17 plays a key role in tumor immunity. There is a reciprocal transformation and antagonistic relationship between Th17 and Treg, and different cytokines maintain the dynamic balance of their quantity and function [57]. Th17 plays an important role in anti-tumor immunity by secreting IL-17 and IFN-γ, expressing CD26, and promoting the activation of CD8^+^ T cells [58–60]. The key mechanism for the differentiation of CD4^+^ naive T cells into Th17 is the activation of transcription factor RORγt via JAK2/STAT3 pathway to initiate the expression of downstream genes related to Th17 function [61].

**Fig. 7:**
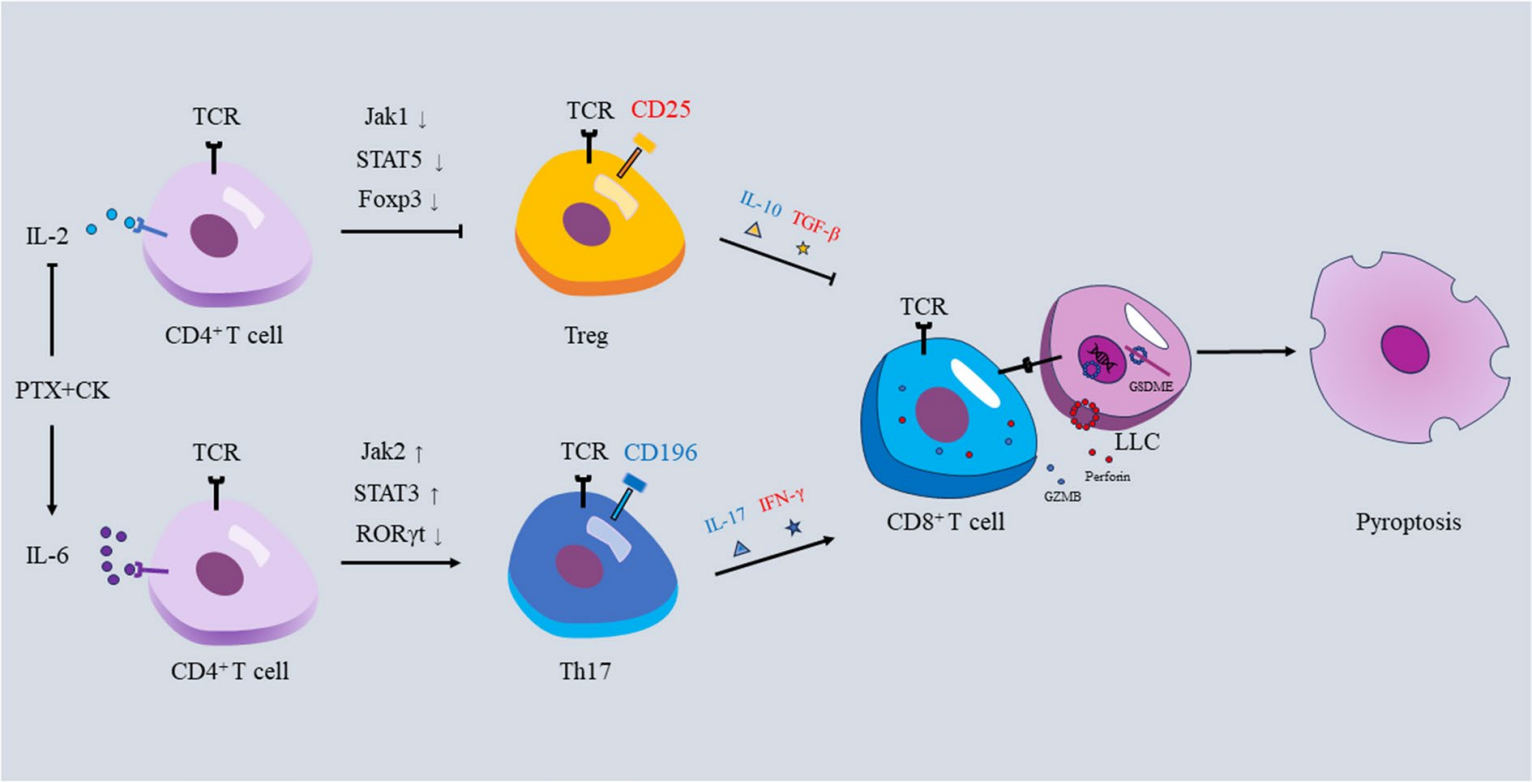
The mechanism of PTX combined with CK

In this study, we combined CK, an immunomodulatory drug, with paclitaxel, a therapeutic drug for advanced NSCLC, to explore its regulatory effect on Treg/Th17 balance, and to provide new ideas for drug development of NSCLC. We first explored the inhibitory effect of PK on the proliferation of NSCLC cells (LLC and A549). Secondly, we co-cultured SPC and LLC to simulate the tumor immune microenvironment to explore the effect of PK on SPC and LLC. As multiple results shown, PK increased the proportion of Th17 and decreased the proportion of Treg in the co-culture system. Cytokines play an important role in the differentiation of Naive CD4^+^T cells. IL-2 and IL-6 regulate the differentiation of Naive CD4^+^T cells into Treg and Th17 by activating JAK1–STAT5 and JAK2–STAT3 pathways, respectively. The increase of Th17 could further increase the number of CTL and promote its infiltration into the tumor. And then, activated CTL induced pyroptosis via the granule exocytosis pathway. Finally, we constructed a C57BL/6 mice LLC xenograft model to verify whether PK exerted its anti-tumor effect by regulating Treg/Th17 balance. We found that PK significantly inhibited the growth of tumor and the mechanism was consistent with the in vitro results. Both in vitro and in vivo results proved that PTX combined with CK could affect the immune microenvironment by regulating the balance of Treg/Th17 (Fig. 7).

Studies have shown that IL-6 played a key role in the differentiation of naive T cells into Th17 [62–64]. However, some studies also demonstrated that IL-6 can promote proliferation and invasion of tumor cells [65–67]. IL-6 is required to bind to IL-6R, which is only expressed in a few types of cells, such as hepatocytes, some leukocytes and epithelial cells [67]. IL-6R expression was examined in SPC and LLC in the present study and was found to be very little expressed in LLC. Therefore, although the concentration of IL-6 in the system was increased, it did not promote the proliferation of LLC. At the same time, our study has some limitations. We demonstrated that PK could regulate Treg/Th17 balance by affecting IL-2 and IL-6, but the regulated mechanism of IL-2 and IL-6 secretion was not explored. Because the SPC contains many kinds of cells, so it is difficult to find the cause of the increased secretion of IL-6, but we hypothesized that IL-6 secreted from macrophages. Furthermore, we did not investigate whether PK could regulate Treg/Th17 balance in other cancers. Moreover, we selected the mouse-derived SPC and LLC for co-culture experiments, however, we did not repeat these experiments in human-derived cells.

Generally speaking, we found that low-concentration PTX combined with CK could exert anti-tumor effect by regulating the tumor immune microenvironment. The clinical treatment guidelines for non-small cell lung cancer (NSCLC) have listed paclitaxel as the first-line chemotherapeutic drug for advanced NSCLC, the widely accepted mechanism is that it binds to tubulin to cause cell mitotic arrest and eventually lead to cell death. However, PTX still has some drawbacks that limit its use. In view of the unique immunomodulatory effect of ginsenoside CK, we tried to combine CK and PTX to study its effect on the immune microenvironment. We demonstrated that low-concentration PTX combined with CK exert anti-cancer role by regulating Treg/Th17 balance and activating CTL. Our study expands the application scope of PTX and provides a new idea for the development of immunotherapy drugs for NSCLC.

## Conclusions

We found that PTX combined with CK can regulate the balance of Treg/Th17 in SPC, increase the number of CTL, activate CTL and play anti-NSCLC role, which provide a new idea for the treatment of advanced NSCLC patients.

## Data Availability

All data associated with this study are present in the paper. Any information for this study is available by contacting the corresponding authors upon reasonable request.

## Abbreviations

CK: Ginsenoside compound K
CTL: Cytotoxic T lymphocyte
Foxp3: Forkhead box protein P3
ICIs: Immune checkpoint inhibitors
IL-17: Interleukin-17
IFN-γ: Interferon-γ
JAK: Janus kinase
LLC: Lewis lung cancer cells
MDSCs: Myeloid-derived immunosuppressive cells
NSCLC: Non-small cell lung cancer
PTX: Paclitaxel
PBMCs: Peripheral blood mononuclear cells
PK: PTX combined with CK
RORγt: Retinoic acid receptor-related orphan receptor γ
ROS: Reactive Oxygen Species
STAT: Signal transducers and activators of transcription
SPC: Mouse spleen lymphocytes
TAMs: T tumor-associated macrophages
TME: Tumor microenvironment
Tregs: Regulatory T cells
Th17: T helper cell 17
